# Mouth Microbes

**Published:** 1888-07

**Authors:** R. L. Cochran


AMERICAN JOURNAL

—OF—

DENTAL SCIENCE.

VOL. XXII. THIRD SERIES—JULY, 1888. NO. 3.

ARTICLE I.

### MOUTH MICROBES.

### DANGERS OF INFECTION IN DENTAL PRACTICE.

[Read by Dr. R. L. Cochran, before t e Indiana Dental Association.]

*Mr. President, Ladies and Gentlemen:*—I have chosen for my text, "Are the natural Teeth saved at the expense of Health and life?" I fear many times they are. Not for the want of a proper filling material nor the lack of skill-ful manipulation; nor is there a Dr. Sexton in it, poor deluded fellow, but my friends, the great cause is in the step taken after your patient is in the chair, and that step is want of order, method, cleanliness—and the greatest of these is cleanliness. Order, gentleman, is the first law of heaven, method is an absolute necessity for the extermination of evil, and cleanliness second only to Godliness, by which the natural teeth can be saved and our patients not suffer in health and perhaps life. My claim is made upon the ground word of the germ theory, to make a broad assertion, and I am almost persuaded to believe true, the dental chair is a positive source of contagion. In my effort to sustain this, I ask your honest, earnest consideration. That germs do exist none can rightfully deny, and that pathogenic germs work destruction to human organism none who read and reflect will doubt. That the germs is not a part of our birth is an established fact, but that we are born with organs that may become favorable soil for the home of the microbe we feel well assured. That the seed of suffering and death are sown we think has been proven beyond a question, and if sown it is our duty as physicians to look well into the whys and wherefores. Why do the germs exist and wherefore shall we look for power to battle against them? Why the pathogenic germs exist I cannot tell; as to the battle man must be the ruler and the microbe must submit. The microscope, the grandest of all known instruments, has given us the knowledge of the organism, and man's intelligence is fast finding the road to their destruction. We may not be able to destroy all pathogenic germs in the world, but we will in time find as sure a specific as has been found for Anthrax and several other diseases of germ origin. The pathogenic germ is man's enemy. The benign germ, his friend. Bacteria are necessary, in fact our farmers would have a waste desert without them. Thousands and millions of bacteria occupy different parts of our bodies and undoubtedly for good. Even the eye is a home for them, and digestion is certainly dependent upon the family of benign germs.

So much for our friends; now for our foes. There is no part of science more interesting or of so great importance to-day, as the study of pathogenic germs. So full of positive concern to all mankind, it is strange indeed that progress has been so slow in this department of science, on which health and life depend. In 1679 a German first saw bacteria and now, after more than two hundred years we are beginning to see the cause of contagion and how easy it is to transmit a pathogenic germ that may mean suffering and even death. It is through the work of germs that milk, fruit and vegetables sour,—butter spoils, and flesh and water become tainted. This is not all, for living plant, and, in fact, all there is of life, is at the mercy of the bacteria family. If bacteria exist in the bodily excretions, the armpits and unwashed feet, what must be the condition of an uncleanly mouth, or the mouth of the germ stricken individual? Simply a bacteria hot house. The fluids of the mouth in composition and temperature are favorable as a culter medium, that is, they contain the elements and at the temperature to encourage their existence. Decayed teeth, foul breath, and bad stomach are the small evils, while lowered vitality and death the final result. I say death because it is not only the benign germ that is found in the mouth, but the genuine pathogenic germ. Is it unreasonable to claim that here may be found the bacillus tuberculosis, the germ of syphilis and undoubtedly many we know not of? When we think that fifty millions may occupy a apace not larger than the period used in printed matter, is it any wonder these small but mighty beggars go unnoticed until they have become an army in numbers, for you know they have a wonderful power of increase? It is stated on good authority that started with one in a favorable medium, it may divide into two in an hour, four in two hours, eight in three hours, and in twenty-four hours will amount to millions. You can from this have some idea of their power of multiplication. They seem to have the live-forever quality and never grow old nor pass into second childhood. Bacteria as a rule are colorless, in fact so they have every advantage over us. We are in the dark until that heaven sunlight the microscope comes to our aid. To be seen with satisfaction they require an amplification of 1,000 diameters; an ordinary man magnified with such power, if standing, his head would be a mile above the ground he stood on. It has been stated the man is safe who serves the best God he knows of, so perhaps the dentist and general practitioner are excused for the destruction they have sown, from the fact that the microbe in size and color was beyond the reach of his unaided sight. But, gentlemen, no excuse can be offered now pathogenic germs exist. All can see them who will take the pains, and careful work will demonstrate their power to multiply and destroy. While it is best to be conservative in all things, yet we must not stand still when health and life are in jeopardy. Microscopist do not claim bacteria as the cause of all disease, yet through the labors of a few devoted to the greatest of all instruments, we are constantly being shown disease after disease of parasitic origin, till we are almost ready to believe this family of dwarfs are at the bottom of all sin, sickness and death, and not Mrs. Eddy's idea of error: nor do we need blinded faith or the supernatural to arrive at an intelligent and positive position. The pathogenic germ is not a modern invention. As far back as history carries us we find this invisible hand at work only under a different name, at one time thought to be the anger of the gods, again a visitation for unfaithfulness, while in truth it was the same enemy of man, pathogenic germs. Had the men of those frightful periods been microscopists instead of being blinded by superstition, thousands of lives could have been saved. But we need not go back into history; the ignorant and weak of to-day look upon these visitations as the "hand-writing on the wall;" the greater the death rate the louder they think the call, when instead of ascribing the calamity to the supernatural, if they would take hold upon the tangible and searchable, use intelligence and the microscope, they would find cause, effect and remedy in this world within our reach. The foes are no longer hidden.

Day by day brings to us knowledge of a new field the microbe is working. The first septic diseases scientifically traced from beginning to end was the silk worm disease in France in 1865 to 1867. Following this came Dr. Koch's discovery of bacillus, anthracis, then the Listerian method, or antiseptic surgery. In 1882 Dr. Koch gave to the world the startling news that he had traced tuberculosis to the bacterial family. That the bacillus of tuberculosis is the only cause of consumption is now an ocular demonstration and not a theory of which any one in doubt can ascertain with a good microscope and technical skill. Here, gentlemen, is the germ to which I wish to call your especial attention. I am well satisfied there are others that should engage our attention, but to the bacillus tuberculosis I wish to direct you at this time. Understand that this, one of the smallest of all living things, does not come into existence spontaneously, nor does it develop in nature outside of the living body. When we become educated as to the working of this bacillus and understand it is common to man and some domesticated animals, and that it can be transmitted from one te another, then can we hope for prevention instead of cure. None are born with tuberculosis, and what we understand as hereditary or predisposition, is that the organs may, under favorable conditions, become a fruitful soil for the bacillus tuberculosis. These living things are endowed with the peculiar quality we call life, and reproduce their kind, although they have no means of themselves to move from place to place, but are carried by the air, or in the food, or from one mouth to another. We are forced by necessity face to face with this enemy of health and peace, and it is simply a matter of their power to attack and our power of resistence, or in other words, if at birth we are endowed with organs proof against the bacillus, of course not including accident, man is most wonderfully constructed and balanced. So true is the balance that often without our knowledge it is thrown out, and who knows, when once out of balance, the invitation is not to some parasite to enter and play havoc. In my opinion, nature would soon right herself were it not for this everpresent devouring element.

Professor Burrill, in his address before the American Society of Microscopists, speaking of tuberculosis, said: "As we come to know more about the various methods by which the virus is conveyed, we shall be better able to escape its contagion." He said further, "that it had been thoroughly demonstrated that the bacillus may be conveyed through the food." That tuberculosis is infectious to those predisposed must be answered in the affirmative.

Although very much light has been thrown on the etiology of tuberculosis by Koch and others, yet there are some who claim unhygienic conditions, physical structure and heredity the only cause. This claim is certainly incorrect. The following is taken from a clinical lecture. A tuberculous man married a young woman with no previous tendency to tuberculous; the man died and the wife became phthisical. She remarried a man who likewise had no phthisical taint; they both died of phthisis. A niece of the woman contracted the disease in nursing her aunt, then married and her husband was in turn attacked by phthisis. Again, a girl returned from school in consumption; on her death her room and clothes passed to her sister, who died of the same disease. A third sister died under like circumstances. As their parents survive them, it is clear that the disease was not due to heredity.

Dr. Trudeau, in the *American Journal of the Medical Sciences*, gave the result of three experiments, which I here quote in full:

"Fifteen rabbits were used, divided into three lots. Five rabbits were inoculated with a pure culture third generation of the bacillus tuberculosis, and deprived of exercise, fresh air and light, with food reduced in quantity. Five more rabbits were placed in a hole ten feet deep, so damp that the box in which the rabbits were confined was always wet. The hole was covered with boards and earth, so they had neither light, fresh air nor exercise, and but a small potato each day. While living in a damp, unhealthy atmosphere, these five rabbits were healthy and removed from germ infection. Five more rabbits were inoculated the same as the first five, and then set free on an island where they had plenty of sunlight, good air, exercise and healthy food, the natural essentials to fight this deadly germ." We learn from these experiments that all of the first five died within four months with lungs extensively diseased. The five in the dark, damp hole, without inoculation and removed from germ infection, were living after four months, and when killed the lungs and surroundings were in a healthy condition. Of the five inoculated rabbits set free, one died within a month with lung disease. The other four entirely recovered from the inoculation, and when killed, four months after inoculation, all the organs were found in a healthy condition. You can readily see, gentlemen, from these experiments, that the bacillus tuberculosis is not developed outside of the living body, and that tuberculosis is entirely dependent upon the working of this microbe. There is no doubt but that the majority of mankind are constantly fighting a battle with the bacillus tuberculosis inoculated by food, air and the dentist, and when inoculation occurs through the agency of the dentist, a fatal result is almost certain, while the germ introduced by food absorption or breathing may not result in phthisis. Dr. Koch has proven that animals fed upon tuberculous food that resisted the attack of the bacillus, yet by direct inoculation soon died of phthisis, So with man; he may resist the germ in his food or that he breathes, but when direct inoculation by means of an instrument in the hands of a dentist it is a certain fatality.

Fortunately, the majority by their very nature and lung condition are sufficiently strong to throw off the deadly bacillus; but where there is a predisposition and direct inoculation occurs, tuberculosis is a certainty. Pathogenic germs require proper conditions of temperature, light, air, etc. Lungs must be by nature or accident a fruitful soil for the bacillus, or nature gains the victory over her unseen foe. I say conditions must be favorable. As an example: Pasteur only succeeded in inoculating chickens with bacillus anthracis after reducing their temperature by holding them in cold water. We also know that animals, as well as the higher type of creation, who live a natural out-doors life on proper food are as a rule free from phthisis. On the other hand over seven millions of our people are yearly afflicted, and possibly one-half die, of tuberculosis, a great many of whom might be saved by proper sanitary measures on the part of the people and order, method and cleanliness on the part of the physician.

We, as physicians, have undoubtedly been sowing the seed year after year. Yes, I admit that is saying a good deal, but stop and think. What is the condition of the substance in which the bacillus tuberculosis is found? A thick, ropy, tenacious mucus, and it must pass through the mouth, and its nature will cling to the mouth, not to say the rough surface of the teeth. Here it lies, the mouth offering it every advantage for its existence. It is not unreasonable to claim that in consumptive persons the substance thrown off the lungs, highly charged with the germ, may cling to and remain about the teeth, and in operating upon those teeth your instruments, mouth-mirror, fingers, in fact everything used about that mouth are very liable to carry away on them the secret foe hidden from the unaided sight. Your next patient, although free from lung complication, yet has the predisposition, or I should say the lungs in such condition as to offer the bacillus a working field; if in such condition what must we expect? Nothing short of inoculation, which in from three to six months will seal the fate of the persons who had confidence in your ability to not only care for the teeth but protect him or her from contagion while being operated upon. Yet certainly do not doubt the existence of the bacillus tuberculosis, and if it does exist you cannot question the possibility of its presence in the mouth of the consumptive. If in the mouth, you dare not deny the probability of its being on your instruments, your rubber dam, your mouth mirror, about your finger nails and everything used in that mouth. If on these articles you must acknowledge when placed in the mouth of your next patient if susceptible to the influence of this germ inoculation is almost certain to occur. This, gentlemen, seems to me to be a fact, cruel as it is, but I am happy to say that the bacillus tuberculosis never need be transmitted from one mouth to another. We are exceedingly fortunate in this particular. Various substances and methods are used to destroy them. The most certain of which is *flame heat* but as flame heat cannot be used on mouth mirror and such articles I present the following table prepared in 1882 by Jalan de La Croix. It shows the number of parts of water to one of the substance which simply permits bacteria to develop.

| | |
|---|---|
| Alcohol, one in . . . . . . . | 30 |
| Eucalyptus, one in . . . . . . | 308 |
| Carbolic Acid, one in . . . . . | 1002 |
| Thymol. one in . . . . . . | 2229 |
| Oil of Mustard, one in . , . . . | 5734 |
| Salicylic Acid, one in . . . . . | 7677 |
| Bi-Chlo-Mercury, one in . . . . | 8358 |
| Sulphuric Acid, one in . . . . . . | 16.782 |
| Iodine, one in . . . . . . | 20.020 |

So with flame heat and the substances named in this table we have the means at hand to destroy this mighty ruler, and if faithful to our trust not a score can be placed against us. I feel sure in the near future it will be greatly to the loss of the physician who neglects to use antiseptic treatment of instruments, appliances and everything used about his patient even to his hands. The value of such a course can be better appreciated after consulted the report from the Maternity hospital of New York.

I will here give you the course of protection I have been following for some time, and if there is a better or more certain method to remove my trusting patient from the danger line, I shall be pleased to know it. First my rooms are daily aired for at least thirty miuutes, no matter how cold. As soon as my patient is in the chair, I wash my hands with soap and clear fresh water, and then rub about the finger nails a solution of one in five hundred Bi-

Chlo Mercury and glycerine. Never use the same water for the second washing. This insures to my patient clean hands and free from germ infection. My operating chair is absolutely clean and free from dust, beaten out every morning; never allow the same napkin on the head rest for the second person. Use the fountain spittoon if possible, if not, then wash out the spittoon with Bi-Chlo Mercury one in five hundred every night and place out of doors over night. I think the spittoon a great evil unless strictly and carefully taken care of; simply washing it out will not do. If it has been used by a tuberculous person the sputa will stick to the sides of the vessel and the next day from a little jar will rise in the shape of dust to be inhaled by your patient or yourself. So the absolute necessity of using a powerful germicide. Never allow bits of cotton, bibulous paper, spunk or the like to remain on the floor about your chair, or in the office. Have a waste basket, put everything of the kind in it and have it carried out every evening, or better put it in the stove. When you take up your mouth mirror wet a corner of a napkin with a 1000 solution of Bi-Chlo Mercury and wash the glass, handle and all. Your instruments of every discription you have ready for use out of sight in tight drawers; after using them you or your assistant should give them thorough attention and destroy every living thing on them. I use and advise a large bunsen burner and every instrument that is touched, good, bad and indifferent, must pass through at least five hundred degrees of flame heat; 212 degrees it is said will destroy the germ, but five hundred flame heat I am sure will. My instructions to my assistant is if she finds an instrument out of place, take it for granted it has been used and pass it through the flame before returning it to place.

Your chip blower and syringe should have the same treatment. Rubber dam should never be used the second time, not even for the same person and don't use the rubber band to hold the dam in place, for fear of scalp trouble; better punch a hole in two corners and use a linen thread tied behind the head, it is perfectly clean and more comfortable to your patient. If you have material on bracket that has not been about the patient's mouth or handled by you while waiting on the person let your assistant put it away or wash your hands in the mercury solution before you touch it. Impression cups should always be passed through the flame after, or just before using. To be safe, and honest to your trusting patient, never use anything until it has been passed through the flame or treated to the mercury solution.

In conclusion let me say be over cautious rather than under cautious; no one ever suffered from a dentist being too careful and don't be afraid of the profession running away with the bugs; better run away with them, than have them run away with our patients.

I am under particular obligations to James Patterson, Esq., of Burlington, Iowa, a gentleman of culture who has given almost his exclusive time for the past ten years to the study of bacteria, and who is the proud possessor of as fine an instrument as there is in the country, with whom I hope to continue the good work until we prove beyond a doubt the claim I have made.—*Southern Dental Journal.*

---

ARTICLE II.

